# Necessity and Readiness for Smoking Cessation Intervention in Dental Clinics in Japan

**DOI:** 10.2188/jea.JE20110038

**Published:** 2012-01-05

**Authors:** Miki Ojima, Takashi Hanioka, Hideo Tanaka

**Affiliations:** 1 Department of Preventive Dentistry, Graduate School of Dentistry, Osaka University, Osaka, Japan; 2 Department of Preventive and Public Health Dentistry, Fukuoka Dental College, Fukuoka, Japan; 3 Division of Epidemiology and Prevention, Aichi Cancer Center Research Institute, Nagoya, Japan

**Keywords:** dentist, patient, health care survey, smoking, smoking cessation

## Abstract

**Background:**

The necessity and readiness for smoking cessation intervention in dental clinics was assessed by investigating smoking status and stage of behavior change in patients and the attitudes of dentists toward the effects of smoking on their patients, respectively.

**Methods:**

A self-administered questionnaire was mailed to 1022 dentists randomly selected from the Japanese Dental Association database. The questionnaire survey consisted of 1 section for dentists and 1 for patients aged 20 years or older and was scheduled to be completed at the dentists’ clinics on a designated day in February 2008.

**Results:**

The response rate to the questionnaire was 78.2% from among target dental clinics and 73.7% and 74.7% for patient and dentist questionnaires, respectively. Data from 11 370 patients and 739 dentists were analyzed. The overall smoking prevalence among the patients (25.1%) was similar to that reported by the National Health and Nutrition Examination Survey, and young female patients had a markedly higher smoking prevalence. More than 70% of patients who smoked were interested in quitting. Although the prevalence of current smoking among dentists (27.1%) was significantly higher than that reported among Japanese physicians (15.0%), approximately 70% of dentists were concerned about the effects of smoking on patient health and prohibited smoking inside their clinic.

**Conclusions:**

Many smokers who were interested in quitting, particularly young women, visited dental clinics, and most dentists believed that smoking was harmful for their patients. These results indicate that smoking cessation intervention in dental settings is necessary and that dentists are ready to provide such interventions.

## INTRODUCTION

Smoking is the most important preventable cause of morbidity and mortality. The prevalence of metabolic syndrome is higher among Japanese male smokers than among their nonsmoking counterparts.^1^ The estimated population-attributable fraction of all-cause mortality due to smoking among Japanese aged 40 to 79 years is 27.8% in men and 6.7% in women.^2^ In Japan, the prevalence of smoking in men is the highest among industrialized countries, whereas that in women is low but has recently increased. Current smoking patterns indicate that comprehensive tobacco control programs should be implemented to reduce the public health burden of smoking-related diseases, which will persist over the next several decades if necessary measures are not taken.^3^

Health professionals have a prominent role in tobacco control.^4^ They interact with smokers when tobacco users are most open to health advice and help them quit smoking through the services they provide in their daily practices. Many studies have shown that behavioral counseling and pharmacotherapy by health professionals are effective in managing nicotine dependence.^5^^–^^7^ Treatment delivered by clinicians in different specialties increased abstinence rates and was more effective than interventions delivered by clinicians in a single speciality.^8^ In short, a multidisciplinary approach is required to identify smokers and treat nicotine dependence.

Dental clinics are expected to have a unique role in creating strategies for smoking cessation intervention.^9^ Oral screening and patient education have always been an important part of routine dental practice. Dental visits therefore provide dental professionals with frequent opportunities to educate their patients with regard to the effects of smoking.^10^^,^^11^ In addition, many studies have reported the efficacy of smoking cessation interventions in the dental setting (ie, the effectiveness of interventions under ideal conditions). A systematic review revealed that behavioral interventions conducted by dental professionals for tobacco users also increased abstinence rates.^12^ Another study reported that the smoking abstinence rate approximately tripled after 1 year of counseling by dental professionals using pharmacologic aids.^13^

Although the potential and efficacy of smoking cessation programs in dental practice are known, the necessity and readiness for such interventions remain unclear because there are few large-scale studies or healthcare data on such interventions in dental clinics. Such data could be used to evaluate how many smokers visit dental clinics, how many are interested in quitting, and how many can be referred to medical institutions for treatment of nicotine dependence. In addition, there are insufficient data on dentists’ attitudes and concern toward the effects of smoking on their patients, which could affect such interventions in their practice.

The present study assessed the necessity and readiness of dental clinics to execute interventional programs for smoking cessation among patients. The study evaluated smoking status, stage of behavior change, and the level of nicotine dependence among dental patients, as well as smoking status among dentists and their attitudes toward the effects of smoking on their patients.

## METHODS

### Data collection

An economic study was designed and conducted to test the hypotheses that smoking-related dental diseases increase the costs of dental care and that smoking cessation interventions in dental clinics decrease those costs. This survey of dental clinics was conducted as part of that economic study. The survey was conducted at each dental clinic on a designated day during the period from 19 to 22 February 2008. Reminder letters were sent twice to nonrespondents, in May and July 2008. For the survey, 1022 dentists were randomly selected from the list of general dental practitioners registered with the Japan Dental Association (JDA; total membership 65 329) in fiscal 2007. The survey comprised 2 parts: 1 for dentists and 1 for their patients. Each dental clinic received a self-administered questionnaire requiring no name identification, a recommendation letter from the director of the JDA, and a cover letter requesting the return of the completed questionnaire by mail. Patients aged 20 years or older who visited the dental clinic on the designated day were informed of the study protocol by their dentist. Patients who gave informed consent to participate in the study completed the questionnaire while waiting for their appointment. Each dentist was asked to enclose the completed questionnaire in an envelope, seal it, and return it in a pre-addressed stamped envelope. The study protocol was approved by the Ethics Committee of Fukuoka Dental College (Ethics Approval No. 115).

### Measures

The questionnaire included questions regarding the smoking status of patients, their readiness to quit smoking, and nicotine dependence. Smoking status was defined as follows: current smoker (an individual who currently smokes and has smoked more than 100 cigarettes since starting), former smoker (an individual who does not smoke currently but has previously smoked more than 100 cigarettes), and nonsmoker (an individual who has never smoked or has smoked no more than 100 cigarettes).^14^

Stage of behavior change, ie, level of readiness to quit smoking, was assessed using the following stages: (1) pre-contemplation stage with no interest to quit (ie, an individual who is not interested in quitting), (2) pre-contemplation stage with an interest to quit or contemplation stage (an individual who is interested in quitting but is not ready to do so within 1 month), and (3) preparation stage (ie, an individual who is ready to quit smoking within 1 month).^15^^,^^16^ The level of nicotine dependence was estimated on the basis of the Tobacco Dependence Screener (TDS), which comprises 10 questions.^17^ Smokers with a TDS score of 5 or higher were defined as being nicotine-dependent.

Dentists were questioned about their smoking status and their attitude regarding the effects of smoking on their patients. Their smoking status was defined in the same manner as that described for patients. Their attitude toward the effects of smoking on their patients was assessed by their answers to the following questions: “Are you concerned about the effects of tobacco smoke on your patients’ health?” and “What preventive measures against passive smoking do you implement in your offices?”.

### Analyses

For the patient data set, we calculated the prevalence of current smokers and 95% CIs for each sex and each of 6 age groups (20–29, 30–39, 40–49, 50–59, 60–69, and ≥70 years). The prevalence of current smoking among dental patients, according to sex and age group, was compared with that of the community, which was assessed during the National Health and Nutrition Examination Survey (NHNS) conducted in November 2007.^18^ The distribution of stage of change and nicotine dependence among current smokers was compared among age groups within each sex. Smoking prevalence among dentists was calculated for each sex and age group. The prevalence of current smoking among male dentists in 2008 was compared with that among male physicians reported in 2000 and 2008.^19^ The percentages of each answer to the questions regarding dentists’ attitudes toward the effects of smoking on their patients were compared among current smokers, former smokers, and nonsmokers. Crosstab procedures were performed using statistical software (PASW Statistics 18, IBM Corporation, NY). The Z and chi-square tests were used to determine statistical significance. The significance level was set at *P* less than 0.05.

## RESULTS

Among 1022 dental clinics, 799 responded to the survey (a response rate of 78.2%): 753 (73.7%) to the patient survey, 763 (74.7%) to the dentist survey, and 717 (70.2%) to both surveys. Of the collected data for 14 187 patients obtained from 753 dentists, information from 2817 (20%) was excluded because of incomplete responses: 4% for the item on smoking status and 16% for other items. Data for the remaining 11 370 patients were then analyzed (Table 1). The mean number of patients per clinic was 15.7 (SD = 7.6). Of the 763 dentists who responded to the dentist survey, 24 were excluded because of lack of information on smoking status. The remaining data for 739 dentists were analyzed. Analysis of the sex and age distributions revealed that males were predominant (92% males and 8% females) and that very few dentists were aged 20 to 29 years or 70 years or older (Table 2).

**Table 1.** Sex and age distribution of dental patients

	Category	*n*	%
Sex	Males	4853	42.7
	Females	6517	57.3

Age group (years)	20–29	1063	9.3
	30–39	1531	13.5
	40–49	1528	13.4
	50–59	2147	18.9
	60–69	2632	23.1
	≥70	2469	21.7

Total		11 370	100.0

**Table 2.** Sex and age distribution of dentists

	Category	*n*	%
Sex	Males	680	92.0
	Females	59	8.0

Age group (years)	20–29	2	0.3
	30–39	82	11.1
	40–49	253	34.2
	50–59	272	36.8
	60–69	118	16.0
	≥70	12	1.6

Total		739	100.0

Table 3 compares the prevalence of current smoking among dental patients and the NHNS population. The overall smoking prevalence among dental patients was 25.1% (40.4% in men and 13.7% in women), which was similar to that of the NHNS population (24.1%). The prevalences of current smoking among male patients aged 40 to 49 and 50 to 59 years were higher than that reported for the NHNS population (*P* = 0.023 and <0.001, respectively). Among female dental patients, the prevalences of current smoking in the age groups of 20 to 29, 30 to 39, and 50 to 59 years were significantly higher than that reported in the NHNS population (*P* < 0.001, 0.001, and <0.001, respectively).

**Table 3.** Comparison of the prevalence of current smoking in dental patients and the population in a national survey^a^

Sex	Age group (years)	Dental patients		Population of survey^a^ %	*P* value^b^

		*n*	% (95% CI)		
Males	20–29	186/366	50.8 (45.7–55.9)	47.5	0.226
	30–39	334/585	57.1 (53.1–61.1)	55.6	0.490
	40–49	337/628	53.7 (49.8–57.6)	49.1	0.023
	50–59	449/907	49.5 (46.2–52.8)	42.3	<0.001
	60–69	422/1211	34.8 (32.1–37.5)	32.8	0.147
	≥70	234/1156	20.2 (17.9–22.5)	18.6	0.174
	
	All	1962/4853	40.4 (39.0–41.8)	39.4	

Females	20–29	175/697	25.1 (21.9–28.3)	16.7	<0.001
	30–39	202/946	21.4 (18.8–24.0)	17.2	0.001
	40–49	173/900	19.2 (16.6–21.8)	17.9	0.332
	50–59	173/1240	14.0 (12.1–15.9)	9.3	<0.001
	60–69	121/1421	8.5 (7.0–10.0)	7.3	0.091
	≥70	52/1313	4.0 (2.9–5.1)	3.7	0.617
	
	All	896/6517	13.7 (12.9–14.5)	11.0	

Total		2858/11 370	25.1 (24.3–25.9)	24.1	

^a^National Health and Nutrition Survey, 2007.^18^

^b^Z-test for proportion.

Table 4 shows the distribution of stage of behavior change and nicotine dependence among patients who currently smoked. The distributions of stage of behavior change and nicotine dependence differed by sex and age group. The overall distribution was 25.8% in pre-contemplation lacking interest to quit, 66.4% in pre-contemplation with interest to quit or contemplation, and 7.8% in the preparation stage. As compared with men, women had a higher level of readiness to quit smoking (*P* < 0.001). Among men, those aged 60 years or older were significantly more likely to be in the preparation stage as compared with younger age groups, whereas no such difference among age groups was seen in women. The prevalence of nicotine dependence (≥5 TDS points) among dental patients was 65.2% (67.1% in men and 60.7% in women). Men had a significantly higher prevalence of nicotine dependence than did women (*P* < 0.001). Among women, those aged 20 to 39 years had a higher prevalence of nicotine dependence than did older age groups, whereas no such difference among age groups was found in men.

**Table 4.** Distribution of stage of change and nicotine dependence among currently smoking dental patients

	Total	Age group (years)			*P*-value^a^

		20–39	40–59	≥60	
Males					
Stage of change	*n* = 1962	*n* = 520	*n* = 786	*n* = 656	0.022
Pre-contemplation without interest to quit	28.9	29.6	29.3	27.9	
Pre-contemplation with interest to quit ​ or contemplation	64.2	64.8	65.3	62.5	
Preparation	6.9	5.6	5.5	9.6	
Nicotine dependence^b,c^	*n* = 1828	*n* = 499	*n* = 731	*n* = 598	0.082
≥5 points	68.1	64.1	69.6	69.6	
<5 points	31.9	35.9	30.4	30.4	

Females					
Stage of change	*n* = 896	*n* = 377	*n* = 346	*n* = 173	0.260
Pre-contemplation without interest to quit	19.0	18.0	19.1	20.8	
Pre-contemplation with interest to quit ​ or contemplation	71.1	71.1	73.4	66.5	
Preparation	9.9	10.9	7.5	12.7	
Nicotine dependence	*n* = 796	*n* = 349	*n* = 306	*n* = 141	0.019
≥5 points	60.8	65.9	58.5	53.2	
<5 points	39.2	34.1	41.5	46.8	

^a^Chi-square test.

^b^Dependence was assessed by using the Tobacco Dependence Screener (TDS).

^c^The total number of current smokers was 2624 for nicotine dependence, due to missing values for the TDS.

The prevalence of current smoking among Japanese dentists was 25.2% in the present study (27.1% in men and 3.4% in women; Table 5). Approximately 90% of female dentists were nonsmokers. Among current smokers, the highest prevalence was seen in those aged 30 to 39 years (34.1%), followed by those aged 40 to 49 years (28.5%). The prevalence of former smoking among male dentists was 32.6%. The prevalence of current smoking among male dentists in 2008 (27.1%) was significantly higher than that among male physicians in 2008 (15.0%) and equivalent to that among male physicians in 2000 (27.1%).^19^

**Table 5.** Distribution of dentists by smoking status

	Category	Smoking status		

		Current smokers % (*n*)	Former smokers % (*n*)	Nonsmokers % (*n*)
Sex	Males	27.1 (184)	32.6 (222)	40.3 (274)
	Females	3.4 (2)	8.5 (5)	88.1 (52)

Age group (years)	20–29	0.0 (0)	0.0 (0)	100.0 (2)
	30–39	34.1 (28)	18.3 (15)	47.6 (39)
	40–49	28.5 (72)	30.0 (76)	41.5 (105)
	50–59	22.4 (61)	34.2 (93)	43.4 (118)
	60–69	20.3 (24)	24.7 (41)	44.9 (53)
	≥70	8.3 (1)	16.7 (2)	75.0 (9)

Total		25.2 (186)	30.7 (227)	44.1 (326)

Table 6 shows the attitudes of dentists toward the effects of smoking on their patients, according to smoking status among dentists. Two-thirds of dentists answered that they were very concerned about the effects of tobacco smoke on their patients’ health. More than three-fourths of dentists answered that they prohibited smoking inside their clinic. Significant differences in the responses to both questions were observed with regard to smoking status (*P* < 0.001). Current smokers were less likely to be concerned about the effects of tobacco smoke on their patients’ health than were nonsmokers or former smokers. In addition, current smokers less frequently prohibited smoking in their offices than did nonsmokers or former smokers.

**Table 6.** Attitudes of dentists toward the effects of smoking on their patients, by smoking status of dentists

Question and category of response	Total^a^ (%)	Smoking status			*P*-value^b^

		Current smokers (%)	Former smokers (%)	Nonsmokers (%)	
*1. Are you concerned about effects of tobacco smoke on patient’s health?*	*n* = 733	*n* = 185	*n* = 224	*n* = 324	<0.001
Very concerned	66.0	44.9	70.5	75.0	
Somewhat concerned	28.8	45.4	26.8	20.7	
Not concerned	5.2	9.7	2.7	4.3	

*2. What preventive measure against passive smoking do you implement in your offices?*	*n* = 738	*n* = 186	*n* = 227	*n* = 325	<0.001
Smoking is prohibited inside entire clinic	76.8	58.6	81.1	84.3	
Other measures	18.8	36.6	16.7	10.2	
No measures	4.3	4.8	2.2	5.5	

^a^Total number of 733 for question 1 and 738 for question 2, due to incomplete responses on questionnaires.

^b^Chi-square test.

## DISCUSSION

In the present study, we collected data on smoking from dentists and their patients. To the best of our knowledge, this nationwide survey is the first of its kind in Japan. The high response rate of 78.2% suggests that the questionnaire was neither too lengthy nor too difficult to answer and that Japanese dentists were interested in the scope of the study.

The overall smoking prevalence among dental patients was 25%, which was similar to that reported in the NHNS population. There was only a small difference in overall smoking prevalence, despite the fact that among some sex–age groups, there was a clear difference in smoking prevalence between dental patients and the NHNS population, which was presumably due to the difference in age-distribution between the 2 groups. Continuing smokers are expected to receive more dental treatment than nonsmokers because of a higher prevalence of perceived dental needs^20^; however, it has been reported that smokers in the United States are less likely than nonsmokers to visit a dental clinic because of their low awareness of dental health.^21^ Current smokers in Japan are less likely to avoid dental visits than those in the United States.^21^ This inconsistency is likely due to differences in the healthcare systems of the United States and Japan, including issues such as insurance coverage and copayment.^22^

Smoking prevalence in female patients aged 20 to 29 and 30 to 39 years was significantly higher than that in the NHNS population. This might be influenced by the fact that women frequently seek dental care or that young female smokers were more concerned about the esthetic effects of smoking.^23^ As compared with male smokers, female smokers had a higher level of readiness to quit smoking. In addition, younger female smokers had a higher prevalence of nicotine dependence than did older women. The proportion of female patients in their 20s and 30s who sought dental care (22.3%) was higher than that of women in the same age groups who sought normal delivery and puerperal care (5.5%).^24^ Therefore, dental professionals may have the opportunity to examine many young female smokers. The development of a smoking cessation program directed at young women who visit dental clinics could reduce the prevalence of smoking in women, which has been increasing in Japan. Such interventional programs conducted in dental clinics could support young smokers who, due to restrictions based on the Brinkman index (ie, cigarettes per day × years smoked), are mostly not eligible for insurance coverage for treatment of nicotine dependence in Japan.

In both male and female patients aged 50 to 59 years, the proportion of current smokers was higher than that in the NHNS population, possibly due to the marked influence of smoking on periodontal tissue in smokers aged 40 years or older, which invariably compels them to visit dental clinics.^25^ Furthermore, periodontal disease is the most frequent reason for tooth extraction in adults aged 45 years or older.^26^ Smokers are more likely to experience tooth loss than are nonsmokers or former smokers.^27^ In this age group, the general health benefits of quitting smoking are more obvious,^28^ considering that smoking-related systemic diseases generally appear in middle-aged smokers. Smoking cessation activities by dental professionals can enhance public health efforts to reduce smoking-attributable morbidity and mortality.

Among current smokers who visited dental clinics, more than 70% were interested in quitting. This indicates that dentists have frequent opportunities to intervene with patients who smoke and are interested in quitting. However, the percentage of smokers who were ready to quit smoking within 1 month (ie, those who were in the preparation stage) was very low (8%). Therefore, motivational approaches using techniques such as the 5 R’s (relevance, risks, rewards, roadblocks, and repetition)^8^ and motivational interviewing^7^ account for many of the smoking cessation interventions in dental practice. A brief intervention using available resources on the various oral effects of smoking (eg, tooth discoloration), which can visually depict the effects, was effective in educating dental patients who were not ready to quit.^16^^,^^29^ This approach is unique to the dental setting; dental clinics are therefore a health resource for organizing interventional programs for smokers, independently of medical facilities.

More than 60% of current smokers had a TDS score of 5 points or higher for nicotine dependence. This result suggests a strong need for nicotine dependence treatment in the dental setting, using pharmacologic aids. Arranging referrals to medical specialists for smokers willing to quit can increase their chances of quitting. Specialist referral services are available in the United States^30^^,^^31^ and United Kingdom,^32^^,^^33^ although the impact of specialist referral requires further evaluation.^34^

The prevalence of smoking among Japanese dentists (27.1% in men and 3.4% women) was lower than that noted in a survey conducted during the period between 2001 and 2006 (30.2% in men and 10.7% in women), although the response rate for that survey was 36%.^35^ However, the prevalence of smoking was considerably higher than that in some other developed countries such as the United States (6%),^36^ United Kingdom (9%),^37^ Norway (7%),^38^ and Australia (4%).^39^ The high prevalence of smoking among Japanese men partly explains the high overall prevalence of smoking, since about 80% of Japanese dentists are men. A survey of physicians revealed that smoking prevalence among male surgeons and otorhinolaryngologists, whose medical practices have characteristics in common with that of dentists, was once significantly higher than that of other specialties.^19^ Although the JDA announced a Declaration for the Nation’s Dental Professionals to Combat Smoking in 2005, smoking prevalence among male dentists in 2008 was still equal to that of male physicians in 2000. Further measures to promote smoking cessation among dentists are therefore necessary.

Interestingly, despite the high prevalence of smoking among dentists, approximately 70% were very concerned about the effects of smoking on their patients’ health and implemented preventive measures against passive smoking. Presumably, this was because of concern regarding the negative effects of smoking on the outcome of treatments. Another reason could be that dentists frequently see young women and children, who are generally sensitive to passive smoking. The implementation rate of smoking prohibition inside the clinic was higher than that in medical clinics (64%), according to the Survey of Medical Institutions, 2008.^40^ Most dentists seemed to support anti-smoking activities in their practice. However, as compared with dentists who were nonsmokers or former smokers, those who currently smoked appeared to be less concerned about the effects of smoking on their patients. The continued promotion of nonsmoking among dentists is essential to increase the public impact of anti-smoking activities in dental clinics.

This study has several limitations. Regarding the dentist survey, the primary limitation was the representativeness of the sample, because not all dentists are JDA members. However, because 70% of dentists working in medical institutes were JDA members as of 2007, the effect of sampling bias due to participation was estimated to be small. A second limitation was that the prevalence of smoking among dentists may have been underestimated in the present study if most dentists who did not return the questionnaires were smokers. Conversely, attitudes toward the effects of smoking on patient health may have been overestimated. In fact, smoking prevalence among dentists who responded to the follow-up letters was higher than that among those who responded to the initial letters (22.5% for the initial, 34.1% for the second, and 45.5% for the third letters; data not shown), which suggests that smokers were less willing to participate in the study than were nonsmokers. A third limitation was that the exclusion rate for patient data was 20% of the collected data, which may cause selection bias. The sex and age distributions of patients were similar to those reported in a patient survey conducted by the Ministry of Health, Labour and Welfare, Japan, in October 2005.^24^ Therefore, the sample of patient surveys in the present study seems to be representative of dental patients throughout Japan. Information on smoking status was available in approximately three-quarters of excluded data. The overall smoking prevalence in these data was 24.3% (data not shown), which was similar to that of the analyzed data (25.1%). Therefore, bias caused by the exclusion of data would be limited with respect to smoking.

In conclusion, many smokers who were interested in quitting smoking, particularly young women, visited dental clinics. In addition, despite their high prevalence of current smoking, most dentists were concerned about the effects of smoking on their patients. These results indicate that smoking cessation interventions undertaken in dental clinics are necessary; furthermore, dentists have positive attitudes toward such interventions for their patients. The dental clinic is more likely to have a significant public impact as a potential health resource if dentists believe that “dentists should not smoke” as well as that “patients should not smoke.” Further studies are required so as to provide information that will enable dental clinics in Japan to improve their ability in implement smoking cessation strategies for their patients.
